# Accurate Visual Simultaneous Localization and Mapping (SLAM) against Around View Monitor (AVM) Distortion Error Using Weighted Generalized Iterative Closest Point (GICP)

**DOI:** 10.3390/s23187947

**Published:** 2023-09-17

**Authors:** Yangwoo Lee, Minsoo Kim, Joonwoo Ahn, Jaeheung Park

**Affiliations:** 1 Dynamic Robotic Systems (DYROS) Lab, Graduate School of Convergence Science and Technology, Seoul National University, Seoul 08826, Republic of Korea; ywlee0305@snu.ac.kr (Y.L.); msk930512@snu.ac.kr (M.K.); 2 Samsung Advanced Institute of Technology, Samsung Electronics, Suwon 16678, Republic of Korea; joonwooahn@snu.ac.kr; 3 Automation and Systems Research Institute (ASRI), Research Institute for Convergence Science (RICS), Seoul National University, Seoul 08826, Republic of Korea; 4 Advanced Institutes of Convergence Technology, Suwon 16229, Republic of Korea

**Keywords:** visual Simultaneous Localization and Mapping, autonomous parking, AVM distortion error, deep learning, weighted Generalized Iterative Closest Point

## Abstract

Accurately estimating the pose of a vehicle is important for autonomous parking. The study of around view monitor (AVM)-based visual Simultaneous Localization and Mapping (SLAM) has gained attention due to its affordability, commercial availability, and suitability for parking scenarios characterized by rapid rotations and back-and-forth movements of the vehicle. In real-world environments, however, the performance of AVM-based visual SLAM is degraded by AVM distortion errors resulting from an inaccurate camera calibration. Therefore, this paper presents an AVM-based visual SLAM for autonomous parking which is robust against AVM distortion errors. A deep learning network is employed to assign weights to parking line features based on the extent of the AVM distortion error. To obtain training data while minimizing human effort, three-dimensional (3D) Light Detection and Ranging (LiDAR) data and official parking lot guidelines are utilized. The output of the trained network model is incorporated into weighted Generalized Iterative Closest Point (GICP) for vehicle localization under distortion error conditions. The experimental results demonstrate that the proposed method reduces localization errors by an average of 39% compared with previous AVM-based visual SLAM approaches.

## 1. Introduction

One of the important technologies for the commercialization of fully autonomous parking is to accurately estimate the pose of the vehicle. Simultaneous Localization and Mapping (SLAM) technology has been developed for localizing the vehicle [1]. Recently, visual SLAM [2], which utilizes cameras as the main sensor, has demonstrated significant advancements in autonomous vehicles, wherein a front camera is commonly employed to estimate the vehicle pose by detecting changes in feature locations or intensity variations of pixels within the image [3]. However, visual SLAM based on a front camera is less suitable for parking scenarios due to the narrow field of view (FOV) of the sensor and a motion bias problem [4] in which the performance of SLAM in forward and backward motion is different.

Instead of a front camera, an around view monitor (AVM) has been also investigated for its application to autonomous parking in visual SLAM. AVM provides a bird’s eye view image using cameras facing in four different directions. Many studies [5,6,7] have applied AVM-based visual SLAM to parking scenarios by taking advantage of wide FOV and no motion bias. These studies have used road-marking information as semantic features to avoid the deformation caused by Inverse Perspective Mapping (IPM). However, inaccurate pose estimation occurs due to AVM distortion error caused by uneven ground and inaccurate camera calibration.

Despite the fact that numerous approaches [8,9,10,11,12] have been explored to achieve accurate bird’s eye view images, they still exhibit several limitations, including limited quantitative comparison between features observed in AVM and features in real-world environments and substantial efforts for collecting data. Alternatively, in [5,6], they avoided the influence of distortion errors by utilizing an additional Inertial Measurement Unit (IMU) sensor based on a pre-built map or leveraging an externally provided High Definition (HD) vector map. The approach of [7] attempted to create an accurate map in real-time using the sliding window fusion technique without additional information, but it exhibited insufficient accuracy in pose estimation for autonomous parking.

This paper proposes an accurate AVM-based visual SLAM against AVM distortion errors caused by an inaccurate calibration without using supplementary information. The main contributions are as follows. First, a novel weighting method is proposed, utilizing a deep learning network, to assign weights to the pixels along the parking lines in the AVM based on the degree of distortion error. Second, a data creation framework is established to minimize the human effort for training the weighting network. Through these two contributions, the shape and magnitude of AVM distortion resulting from inaccurate calibration are readily predicted. In contrast to conventional data creation methods which entail human effort in manually annotating ground truth data for each raw data, the proposed data creation framework automatically generates data by utilizing three-dimensional (3D) Light Detection and Ranging (LiDAR) SLAM. By employing parking line points with varying weights based on the degree of distortion error, the proposed SLAM has shown an improved localization performance with an average reduction of 39% in error compared with the modified approach of Hybrid Bird’s-Eye Edge-Based Semantic Visual SLAM [7] when implemented in various real-world parking lots.

The rest of the paper is organized as follows. In the next section, the background of this paper, including related work and the limitations of Hybrid Bird’s-Eye Edge-Based Semantic Visual SLAM [7], is presented. Then, in Section 3, an overview of AVM-based SLAM using a weight map and novel dataset creation method is described. After describing the experiment’s results and the discussion in Section 4, Section 5 concludes this study and refers to future works.

## 2. Background

### 2.1. Related Work

#### 2.1.1. Visual SLAM

##### Front-Camera-Based Visual SLAM

In the field of visual SLAM, there are various methods using a front camera as the main sensor. These can be broadly categorized into direct and feature-based methods. Direct methods use the raw image, while feature-based methods use only features in the image. Representative direct methods of visual SLAM include Direct Sparse Odometry (DSO), which estimates camera pose by minimizing the pixel intensity difference between two images [13]. ORB-SLAM2 [14] is a well-known feature-based method that estimates camera pose by minimizing the distance difference between matched feature points. Another method, Semi-direct Visual Odometry (SVO), combines the advantages of both direct and feature-based methods [15]. However, these methods are not suitable for autonomous parking. A front camera with a narrow FOV can have challenges detecting changes in the surrounding environment of a vehicle during rapid rotation. Moreover, depth estimation in these methods causes a difference in SLAM performance between forward and backward motion [4].

##### AVM-Based Visual SLAM

Various SLAM studies have proposed utilizing AVM as the main sensor for autonomous parking. Autonomous valet parking-SLAM (AVP-SLAM) [5] estimates the pose of the vehicle by matching current road-marking features with a road-marking map created in the preceding process. AVP-Loc [6] matches current road-marking features to an HD vector map of a parking lot for vehicle localization. In [7], the edge information of both free space and road markings are utilized as input features to estimate the pose of a vehicle using Iterative Closest Point (ICP) [16]. However, the AVM distortion error was not taken into account in these studies. In [5], the inherent inaccuracies caused by the distortion error in the pre-built map necessitated an additional IMU to enhance the localization accuracy. The method of [6] employed an externally provided HD vector map to avoid distortion errors. Although the approach in [7] aimed to create an accurate map in real-time using the sliding window fusion technique without additional information, the construction of maps that include distortion errors negatively affects the localization performance of autonomous vehicles.

#### 2.1.2. AVM Image Enhancement Techniques

##### AVM Image Modification Using Automatic Calibration

Several methods have been proposed for the automatic calibration of AVM images, utilizing extracted feature shapes to achieve accurate bird’s eye views. In [8], a method was proposed to estimate the extrinsic parameters of four AVM cameras using point patterns. The method proposed in [9] aims to calibrate an AVM image by matching the gradient and position of the lane observed from the front and rear cameras with that seen from the left and right cameras. On the other hand, the method proposed in [10] focuses on calibrating the AVM image to ensure the detected parking lines are parallel or perpendicular to the vehicle. However, since these methods only perform relative comparisons between the images of each camera and do not quantitatively compare the AVM image with the real environment, it may not be considered as a complete solution to address AVM distortion errors.

##### AVM Image Generation Using Deep Learning

In recent years, there has been a shift towards deep learning-based AVM image generation methods that use Neural Networks (NN) as viewpoint transformation functions, instead of using homography geometric information. HDMapNet [11] uses a Multilayer Perceptron (MLP) to convert each camera image from AVM into a bird’s eye view, and then creates an AVM image through an attaching process using installed camera locations. Another method, BEVformer [12], uses the Transformer model to create an AVM image without an additional attaching process. However, these methods require a large amount of training data.

### 2.2. Limitation of Hybrid Bird’s-Eye Edge-Based Semantic Visual SLAM

To estimate the vehicle pose without the need for pre-map construction or additional sensors, the algorithm of the Hybrid Bird’s-Eye Edge-Based Semantic Visual SLAM [7] is selected. Instead of using the method directly, it was modified in two aspects to improve the pose estimation performance. First, parking line information is utilized as input data instead of edge information from semantic features and free space. Second, Generalized ICP [17] is used instead of ICP for pose estimation. GICP is a pointcloud registration algorithm developed from ICP that addresses inaccurate correspondences. The modified Hybrid Bird’s-Eye Edge-Based Semantic Visual SLAM is used to examine the impact of distortion errors, resulting from inaccurate AVM calibration, on the performance of AVM-based SLAM. This will be compared with the proposed method in Section 4.

**Figure 1:**
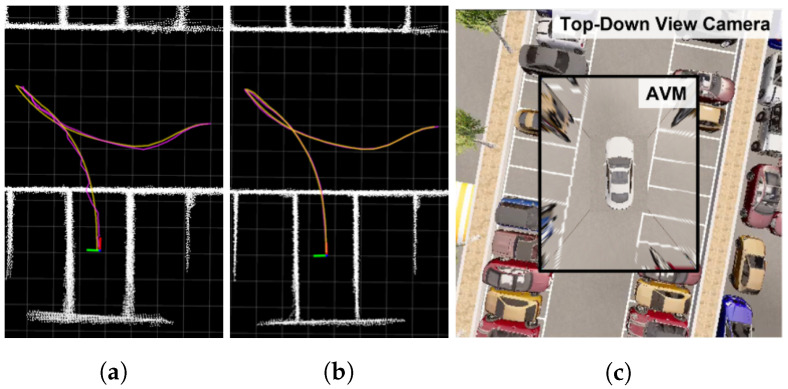
Comparison between Hybrid Bird’s-Eye Edge-Based Semantic Visual SLAM [7] (**a**) and the modified Hybrid Bird’s-Eye Edge-Based Semantic Visual SLAM (**b**). Magenta trajectory is SLAM trajectory, orange trajectory describes ground truth trajectory, and white pointcloud map is global map built by SLAM. (**c**) shows comparison between AVM and top-down view camera in CARLA simulator.

Figure 1a,b show the improved performance of the modified Hybrid Bird’s-Eye Edge-Based Semantic Visual SLAM compared with the original Hybrid Bird’s-Eye Edge-Based Semantic Visual SLAM [7]. To compare the algorithmic performance in the same environment without any disturbance, the CARLA simulator [18], an open-source autonomous driving vehicle simulator, was utilized. The AVM image, as shown in Figure 1c, generated using the actual AVM image generation method, was used.

#### 2.2.1. Avm Distortion Error

**Figure 2:**
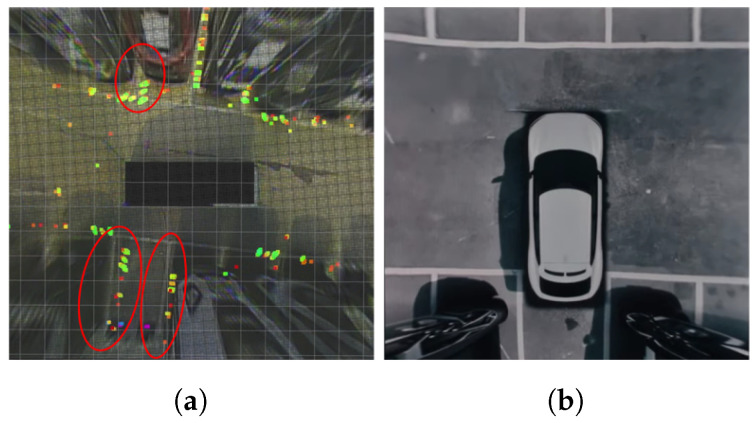
AVM distortion error. (**a**) compares AVM image with parking line points extracted from 3D LiDAR. The red circles in (**a**) shows disparity between the locations of color points from 3D LiDAR and white parking line pixels from AVM. (**b**) shows AVM distortion in commercial AVM.

To assess the AVM distortion error caused by inaccurate AVM calibration, a comparison between the parking line information extracted from the 3D LiDAR and the parking line information of the AVM image (represented as color points and white parking line pixels, respectively, in Figure 2a) was conducted. The region marked with a red circle in Figure 2a highlights AVM distortion error as showing disparity between the locations of two kinds of features from different sensors. The intensity rate and height information of the pointcloud are used for selecting feature points from the raw LiDAR pointcloud and the coordinate system of the AVM and 3D LiDAR were pre-aligned beforehand. Even commercial AVMs are not exempt from distortion. As shown in Figure 2b, despite the even ground, the parking line, which should ideally appear straight, is observed as a curved line in the AVM representation. Inaccurate camera calibration can arise due to errors that occur during the calibration process using calibration targets and software-based automated calibration. Additionally, factors such as manufacturing tolerances, environmental conditions like temperature and vibrations, and sensor performance degradation over time can also contribute to inaccurate camera calibration.

#### 2.2.2. SLAM Performance Comparison in Simulation Environment and the Real World

To assess the impact of AVM distortion errors on SLAM performance, experiments were conducted using the aforementioned modified Hybrid Bird’s-Eye Edge-Based Semantic Visual SLAM [7] in both the CARLA simulator and a real-world environment with an even ground. The AVM image obtained from the CARLA simulator, as shown in Figure 1c, exhibits a lower distortion error compared with the AVM image in the real environment.

**Figure 3:**
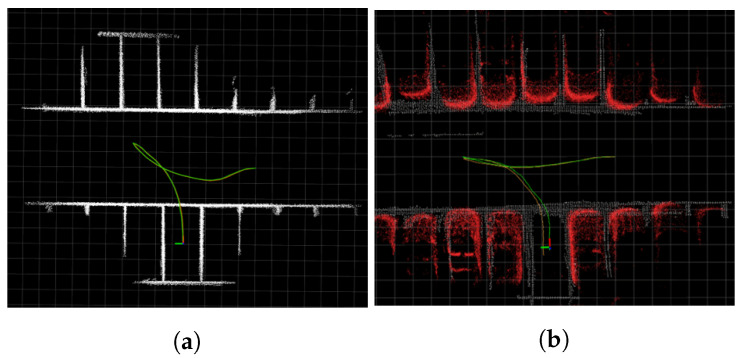
Comparison experiment in CARLA simulator and real world. (**a**,**b**) describe results of modified Hybrid Bird’s-Eye Edge-Based Semantic Visual SLAM in different environments with even ground. Green trajectory is SLAM trajectory, orange trajectory describes ground truth trajectory. A 3D LiDAR SLAM was used as ground truth in the real-world environment. White pointcloud map is a global map built by SLAM. Red pointcloud map in (**b**), marked for visualization, is obstacle pointclouds from 3D LiDAR.

Figure 3a presents the results of the modified Hybrid Bird’s-Eye Edge-Based Semantic Visual SLAM in the CARLA environment, while Figure 3b shows the results of the identical method in a real-world environment, with a higher distortion error compared with the simulator environment. This emphasizes the importance of overcoming the AVM distortion error. Quantitative comparison is presented in Section 4.

## 3. Proposed Method

### 3.1. Framework of AVM-Based Visual SLAM Using Weight Map

**Figure 4:**
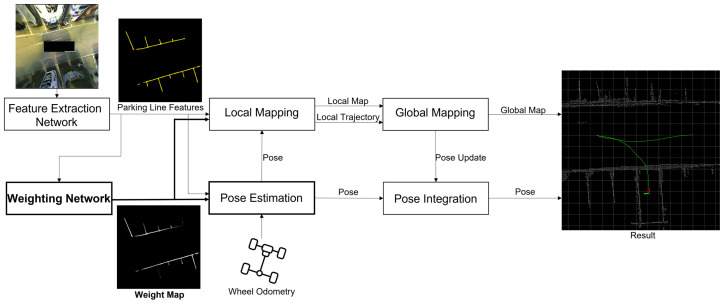
System architecture of AVM-based visual SLAM using weight map.

Figure 4 shows the framework of the proposed method, which extends the approach of the modified Hybrid Bird’s-Eye Edge-Based Semantic Visual SLAM by incorporating a feature weighting network. The current pose is estimated by weighted GICP using parking line pointcloud, predicted weight map, and wheel odometry. The pose at each time step accumulates in the local trajectory, and the parking line pointcloud accumulates in the local map. The estimated poses are updated through matching between the newly created local map and the global map.

**Figure 5:**
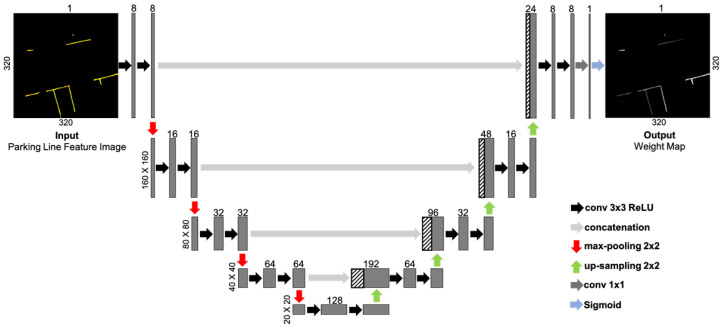
The architecture of feature weighting network.

The feature weighting network receives the parking line information detected from LaneNet [19] as the input and generates a weight map that contains weight information for each parking line pixel. As shown in Figure 5, the network is constructed based on the U-Net [20] architecture, which is renowned for its ability to capture spatial features by combining down-sampling and up-sampling pathways, enabling it to comprehend contextual information. And the size of the U-Net network was reduced for real-time performance by decreasing both the input image size and the number of feature maps used in its layers. Additionally, a sigmoid activation function was added at the end to ensure that the predicted weights fell within the range of 0 to 1. A higher AVM distortion error leads to a lower weight assignment, improving pose estimation performance in the presence of distortion errors.

(1) $$\begin{array}{c} T=\arg\min_{T}\sum_{i}\left(1+w_{i}^{A}\right)\cdot \left(1+w_{i}^{B}\right)\cdot d_{i}^{{\left(T\right)}^{T}}\cdot {\left(C_{i}^{B}+T\cdot C_{i}^{A}\cdot T^{T}\right)}^{-1}\cdot d_{i}^{\left(T\right)}\\ where\;d_{i}^{\left(T\right)}∼N\left(T\cdot b_{i}-a_{i},\;C_{i}^{B}+T\cdot C_{i}^{A}\cdot T^{T}\right)\; \end{array}$$

For pose estimation, the parking line pointcloud converted from parking line information, corresponding to the predicted weight map and wheel odometry information, are used as the input to the weighted GICP, which is an optimization method that minimizes the weighted sum of distance considering the distribution of each point in an associated pair, as shown in Equation (1). In the equation, *T* represents the transformation matrix used for pose estimation. The pointclouds $A={\left\{a_{i}\right\}}_{i=1}^{N}$ and $B={\left\{b_{i}\right\}}_{i=1}^{N}$ are indexed based on their correspondences, where $a_{i}$ corresponds to $b_{i}$. The pointclouds A and B represent the parking line pointclouds at time t and t + 1, respectively. The weight factor *w* is derived from the weight map and is associated with each point *i*. *C* represents the covariance matrices of the points. The term $d_{i}^{{\left(T\right)}^{T}}\cdot {\left(C_{i}^{B}+T\cdot C_{i}^{A}\cdot T^{T}\right)}^{-1}\cdot d_{i}^{\left(T\right)}$ represents the Mahalanobis distance [21], which quantifies the distance between two associated points by considering their distributions. In Equation (1), the Mahalanobis distance is considered as an error. In order to emphasize the errors that need to be minimized, each error is multiplied by the weight of the two points associated with the error. However, when a considerable portion of the parking line points receive small weights, the weighted sum of errors tends to converge to zero before obtaining the optimal transformation matrix. To counteract this effect, the weights were used by adding one to them. Assigning all parking line point weights as 0 results in the weight term becoming 1, thus transforming Equation (1) into an equivalent equation for pose estimation within the modified Hybrid Bird’s-Eye Edge-Based Semantic Visual SLAM, which aligns with the GICP algorithm. Wheel odometry is utilized to project the current parking line pointcloud onto the local map, which helps prevent convergence to a local minimum during the optimization.

The pose update takes into account the relative pose from trajectory between the newly created local map and the global map using weighted GICP. Both the local map and the global map use a grid map representation, where each grid accumulates the weights of the included points. This indicates that grids containing larger values included a greater number of points with less AVM distortion. Additionally, points with weights lower than the heuristic threshold were removed. As a result, the vehicle pose is accurately updated through matching between grid cells with low distortion errors.

### 3.2. Dataset Creation

**Figure 6:**
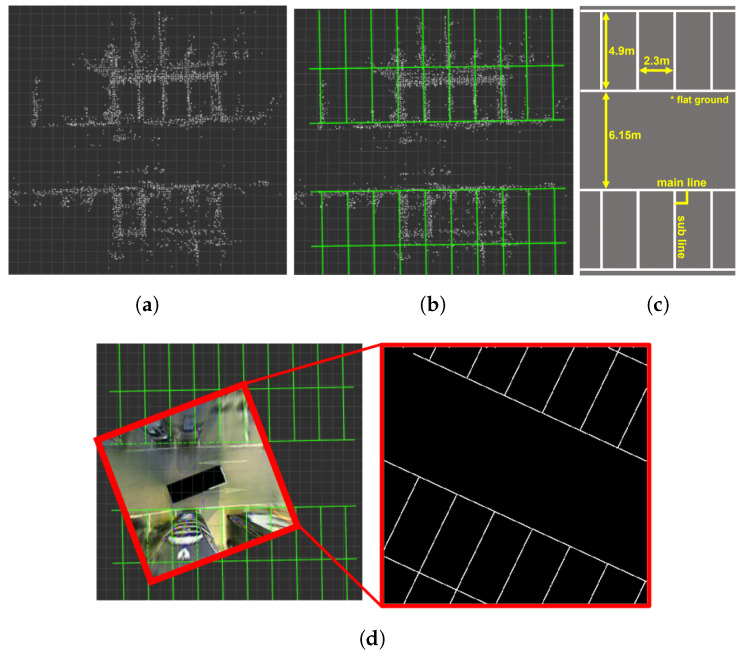
Creating ideal parking line images. (**a**) shows stacked parking line pointcloud map based on 3D LiDAR SLAM. Each white point in (**a**) represents parking line points extracted from 3D LiDAR. (**b**) shows ideal parking line map matched with (**a**). (**c**) shows official parking lot drawing guideline with pre-measurement information. (**d**) shows the generation of ideal parking line images by capturing the ideal parking line map from a series of the AVM viewpoints, which are obtained based on the poses from the 3D LiDAR SLAM.

**Figure 7:**
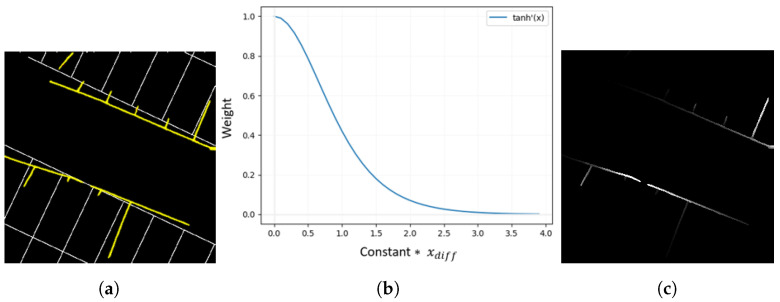
Ground truth creation method. (**a**) shows difference between detected parking lines (yellow lines) and ideal parking lines (white lines). (**b**) describes the hyperbolic tangent derivative function. (**c**) shows the ground truth weight map.

There are two stages to assigning weights to each pixel of the parking line based on the AVM distortion error. The first stage involves creating ideal parking line images using LiDAR data and measurement information (see Figure 6). The second stage assigns weights to each detected parking line pixel by comparing the detected parking lines from the AVM image with the ideal parking lines (see Figure 7).

Figure 6 shows a creating method of the ideal parking line images. During a parking scenario, a parking line feature pointcloud is extracted using the intensity and height information of each point in the 3D LiDAR. These feature pointclouds are accumulated through 3D LiDAR SLAM to construct an initial feature map, as shown in Figure 6a. An ideal parking line map, which corresponds to the green parking lines in Figure 6b, is built in the form of a pointcloud based on pre-measured information and official parking lot guidelines, as shown in Figure 6c. The initial feature map and the ideal parking line map are aligned using GICP. Subsequently, as shown in Figure 6d, the ideal parking line images are generated by capturing the aligned ideal parking line map in the same size as the AVM image from the AVM viewpoints, which are obtained through a series of poses of the 3D LiDAR SLAM used in Figure 6a. The ideal parking line images are utilized to create a ground truth weight map for training a feature weighting model.

**Figure 8:**
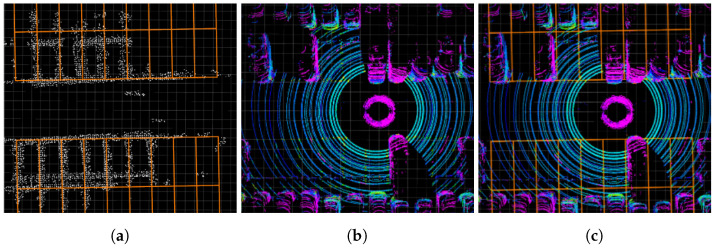
Comparison between ideal parking line map and an actual parking lot. (**a**) shows ideal parking line map (orange lines) matched with stacked parking line pointcloud map (white pointcloud). In (**b**), the pointcloud from the 3D LiDAR is visualized with varying colors corresponding to the point intensity ratio. (**c**) shows the alignment between the ideal parking line map (orange lines) and the parking lines (yellow and green points) in (**b**).

In Figure 8, a qualitative evaluation was conducted to assess the similarity between the ideal parking line map and an actual parking lot. Figure 8b shows the LiDAR pointcloud obtained from the actual parking lot, where different colors are assigned to each point based on their intensity ratio. In Figure 8b, the parking lines are represented by yellow and green points arranged in a grid pattern. Figure 8c demonstrates the alignment between the ideal parking line map and the parking lines in the LiDAR pointcloud, indicating the similarity between the ideal parking line map and the actual parking lot.

The detected parking line information from the AVM image is compared with the ideal parking line image, and a weight is assigned based on the position difference between the detected parking line pixels and the ideal parking line pixels as shown in Figure 7a, which represents the AVM distortion error. In order to accurately calculate the position difference, the detected parking lines and ideal parking lines are classified into main line points and sub line points based on the normal vector direction of each point. Using the NearestNeighbor algorithm [22], the Euclidean distances between the detected main line points and their corresponding ideal main line points, as well as the distances between the detected sub line points and their corresponding ideal sub line points, were calculated. The distances are multiplied by an experimentally determined constant and then incorporated into the hyperbolic tangent derivative function, as illustrated in Figure 7b. Unlike the commonly used min-max scaling function ($\frac{x-min(x)}{max(x)-min(x)}$) in normalization calculations, the hyperbolic tangent derivative function clearly distinguishes points with large distance values from those with small distance values. This enables one to focus the weight assignment on points with smaller distortion errors. Finally, a ground truth weight map is generated as shown in Figure 7c, where weights are assigned along the detected parking lines.

## 4. Experiment and Discussion

### 4.1. Experimental Setup

**Figure 9:**
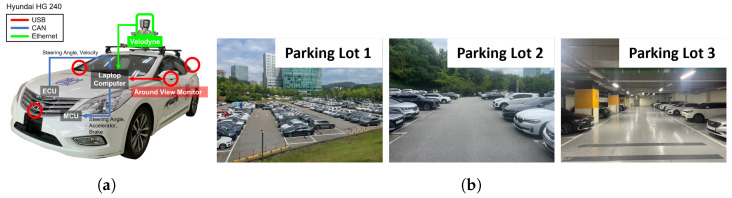
Experimental setup. (**a**) shows autonomous vehicle. (**b**) shows the parking lots used for data collection and experiments. The training data was collected in Parking Lot 1, and the experiments were conducted in Parking Lot 1 (excluding the place where the training data were collected), Parking Lot 2, and Parking Lot 3.

As shown in Figure 9a, the HG 240 vehicle was utilized for data collection and experiments. The AVM was installed by a specialized company, Omniview. The laptop computer used in the experiment has the following specifications: an Intel i9-9900 CPU from Intel, Santa Clara, USA, NVIDIA RTX 2080 GPU from Nvidia, Santa Clara, USA, and 32GB of RAM. The development and visualization tasks were carried out using the Robot Operating System (ROS).

The training data were collected from Parking Lot 1, as shown in Figure 9b, located within the Gwang-Gyo Techno-Valley located in Gwanggyo-dong, Suwon city, at Seoul National University. A total of 2512 data points for training were acquired through various perpendicular parking scenarios with different locations of the goal position relative to the starting point of the autonomous vehicle and different numbers of parked vehicles adjacent to the goal position. The data were categorized into training (nine scenarios) and validation (two scenarios) sets for training the feature weighting model. The weighted mean square error loss was utilized for the loss function, with a training batch size of eight and a learning rate of $1\times 10^{-5}$. The weights in the loss function are assigned to the pixels where the parking lines exist. The dataset is available via the following link: https://github.com/ywlee0305/Accurate-Visual-SLAM-against-AVM-Distortion-Error-using-Weighted-GICP-Dataset/tree/master/Training_Data, accessed on 20 August 2023. The file encompasses raw AVM images, parking line data detected by LaneNet [19], and the ground truth weight maps.

To evaluate the performance of the proposed method, experiments were conducted in three different parking lots, as shown in Figure 9b. The vehicle was parked at an average speed of 2 km/h. The ground truth for evaluation was obtained using a 3D LiDAR SLAM called LeGO-LOAM [23]. The Absolute Trajectory Error (ATE) [24], which is the difference between the ground truth and estimated poses, was used as the performance metric. ATE is calculated with Equation (2).

(2) $$\begin{array}{c} ATE=\frac{1}{N}\sum_{i=1}^{N}∥p_{i}^{\mathrm{estimated}}-p_{i}^{\mathrm{ground}\;\mathrm{truth}}∥ \end{array}$$

where *N* is the total number of poses, $p_{i}^{\mathrm{estimated}}$ represents the estimated pose at time step *i*, and $p_{i}^{\mathrm{ground}\;\mathrm{truth}}$ represents the ground truth pose at time step *i*. The symbol ∥·∥ denotes the Euclidean distance between the estimated and ground truth poses.

### 4.2. Quantitative Comparison between CARLA Simulation and Real Environments

Table 1 presents the quantitative results of the modified Hybrid Bird’s-Eye Edge-Based Semantic Visual SLAM in the CARLA environment and real environment. Table 1 shows that ATE is increased by approximately five times due to AVM distortion errors.

### 4.3. Proposed Method Evaluation and Discussion

**Figure 10:**
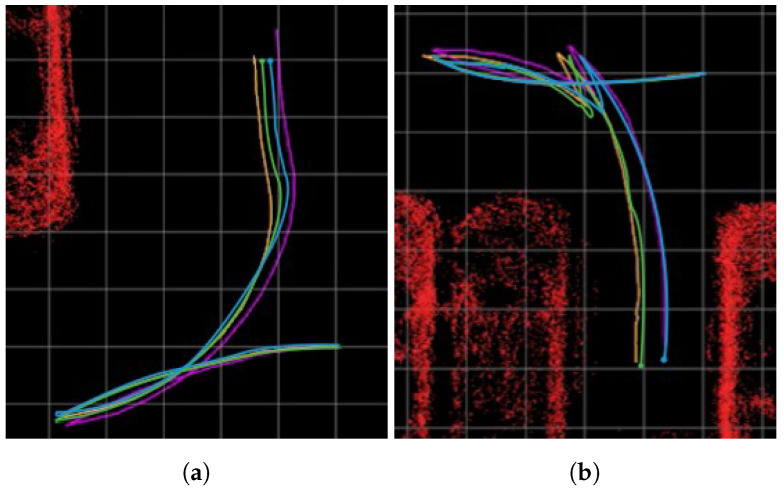
Trajectory comparison between the Particle-Filter-based Semantic Visual SLAM [25], the modified Hybrid Bird’s-Eye Edge-Based Semantic Visual SLAM, and the proposed method in scenario 1 (**a**) and scenario 8 (**b**). Purple trajectories are the Particle-Filter-based Semantic Visual SLAM [25] trajectory, light blue trajectories are the modified Hybrid Bird’s-Eye Edge-Based Semantic Visual SLAM trajectory, green trajectories are the proposed method trajectory, and orange trajectories describe ground truth trajectory. Red pointcloud maps in the Figure 10, marked for visualization, are obstacle pointclouds from 3D LiDAR. The size of each grid is 1 m × 1 m.

The comparison experiments between the Particle-Filter-based Semantic Visual SLAM [25], the modified Hybrid Bird’s-Eye Edge-Based Semantic Visual SLAM, and the proposed method were conducted in various parking scenarios, as shown in Table 2. PF [25] is one of the methods that use a Particle Filter among semantic visual SLAM. Experiments 1 to 6 were conducted at various locations within the parking lot (Parking Lot 1), where the training data were collected. Experiments 7 to 10 were conducted at different parking lots (Parking Lot 2, Parking Lot 3). Table 3 presents the quantitative results of the experiments. In PF [25], particles represent the predicted vehicle poses and are randomly sampled using the constant velocity motion model. Each particle’s weight corresponds to the number of overlapped pixels between the current parking line feature and the previously constructed map. Subsequently, the particle with the highest weight is selected as the vehicle’s pose for the next step. The map was created using parking lines extracted with LaneNet [19], and the map was merged using the selected particle pose information. PF [25] exhibits inferior pose estimation performance compared with the modified Hybrid Bird’s-Eye Edge-Based Semantic Visual SLAM and the proposed method. This drawback arises from inaccuracies in map generation due to AVM distortion errors, leading to decreased accuracy of the highest-weighted particle and, consequently, a less-precise map. Furthermore, the absence of pose updates at the local map level allows errors to accumulate throughout the scenario. The estimated trajectories for each method in scenarios 1 and 8 are displayed in Figure 10. The maximum value of ATE when using the proposed method was reduced by 34.0% compared with when using the modified Hybrid Bird’s-Eye Edge-Based Semantic Visual SLAM. The mean value of errors was reduced by 39.0% and the root mean square error (RMSE) was reduced by 37.9%. The Final Parking Localization Error (FPLE), which represents the localization error of the vehicle after parking, was also decreased in all scenarios. Additionally, a computation load comparison between the proposed method and ORB-SLAM2 [14] was conducted, focusing on assessing the practical usability of the proposed approach. The feature weighting network and the pose estimation in a single iteration of the proposed method exhibit average computation loads of 90 Hz and 23 Hz, respectively, whereas that of ORB-SLAM2 [14] is 18 Hz. This comparison highlights the real-time capability of the proposed method. The entire results are described in Table 3.

**Figure 11:**
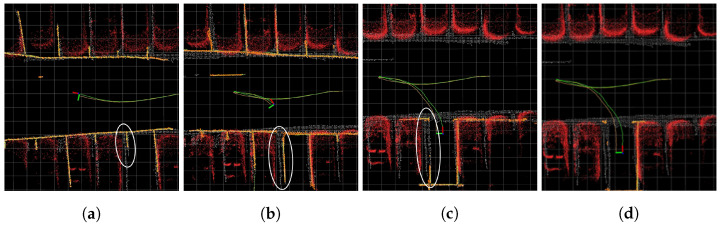
Sequence of the modified Hybrid Bird’s-Eye Edge-Based Semantic Visual SLAM in experiment 6. (**a**–**d**) represent a sequence of snapshots captured during the experiment. Green trajectory is estimated trajectory, orange trajectory describes ground truth trajectory, orange pointcloud describes local map, and white pointcloud map signifies global map built using SLAM. Red pointcloud map, marked for visualization, is 3D LiDAR SLAM obstacles. White circles show the inaccurate mapping process. The size of each grid is 1 m × 1 m.

**Figure 12:**
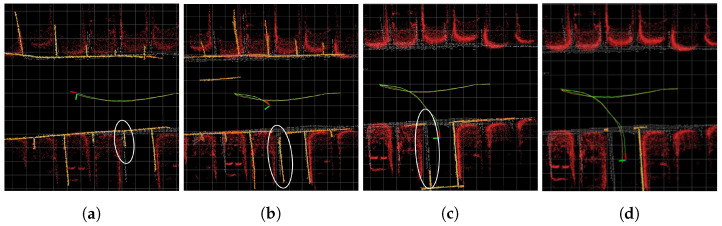
Sequence of proposed method in experiment 6. (**a**–**d**) represent a sequence of snapshots captured during the experiment. Green trajectory is estimated trajectory, orange trajectory describes ground truth trajectory, orange pointcloud describes local map, and white pointcloud map signifies global map built using SLAM. Red pointcloud map, marked for visualization, is 3D LiDAR SLAM obstacles. White circles show the inaccurate mapping process. The size of each grid is 1 m × 1 m.

Figure 11 and Figure 12 show the sequence of experiment 6. As depicted in Figure 11a, not considering the weights of parking line points affected by distortion errors resulted in inaccurate pose estimation, leading to the inaccurate global map. Subsequently, as shown in Figure 11b,c, pose updates using the inaccurate global map failed to correct the poses accurately. The white circle in Figure 11 indicates an inaccurately constructed global map. In contrast, Figure 12a demonstrates that by focusing on parking line points with minimal distortion errors, the accurate pose estimation led to an accurate global map. Consequently, as illustrated in Figure 12b,c, incorporating the global map composed of points with low degree of distortion errors enabled more precise pose updates. The white circle in Figure 12 highlights a more accurately constructed global map compared with Figure 11. Moreover, the final pose of the vehicle depicted in Figure 12d shows better alignment with the ground truth compared with the final pose of the vehicle shown in Figure 11d.

In Table 3, the proposed method in experiments 9 and 10 did not exhibit a significant reduction in ATE compared with the results obtained from the modified Hybrid Bird’s-Eye Edge-Based Semantic Visual SLAM. This can be attributed to a wall at the rear of the goal position and a limited visibility of parking lines in the AVM due to parked vehicles around the goal position. As a result, there were fewer parking line points with minimal distortion errors, leading to less influence of the predicted weights on the pose estimation.

## 5. Conclusions

This paper proposed an AVM-based visual SLAM framework for autonomous parking that addresses the challenge of AVM distortion errors. By incorporating a feature weighting network, weights are assigned to parking line features based on the degree of distortion. A novel dataset creation method that minimizes human intervention was proposed, and the ideal parking line map constructed using this method was validated by comparing it with the actual parking lot. Through experiments, the proposed method reduced the error by an average of 39% compared with the modified Hybrid Bird’s-Eye Edge-Based Semantic Visual SLAM. The proposed method also enhances the accuracy of the estimated vehicle pose after parking.

However, the proposed method showed less improvement in environments where features are sparsely detected due to walls and parked vehicles, compared with environments with more detected features. Additional data training specific to environments with different shapes, such as diagonal parking lots, is required to effectively apply the proposed method. To overcome these limitations, exploring different features that can be utilized with parking lines is needed. Further evaluations in various parking scenarios are required to generalize the proposed method. Conducting experiments in scenarios including dynamic obstacles and partially occluded parking lines is also essential. Moreover, performance evaluations in environments with distortion errors caused by uneven ground are also necessary.

## Figures and Tables

**Table 1:** Result of absolute trajectory error (ATE) in CARLA environment and real environment. RMSE means root mean square error. The minimum values for both environments are small and are not specified in the table.

Environment	Max [m]	Mean [m]	RMSE [m]
CARLA	0.117	0.053	0.061
Real	0.549	0.260	0.299

**Table 2:** Experiment scenarios. The scenarios were categorized based on the experiment location, the number of adjacent parked vehicles near the goal position, the position of the goal position relative to the initial pose of the autonomous vehicle (whether it is on the left or right side), and the number of cusps indicating the switching points between forward and backward movements of the autonomous vehicle.

Case	Experiment Place	The Number of Adjacent Vehicles	The Location of the Goal Position	The Number of Cusps
1	Parking Lot 1	0	right	1
2	Parking Lot 1	0	left	1
3	Parking Lot 1	0	left	3
4	Parking Lot 1	1	left	1
5	Parking Lot 1	1	left	3
6	Parking Lot 1	2	left	1
7	Parking Lot 2	1	right	1
8	Parking Lot 2	2	left	3
9	Parking Lot 3	2	right	3
10	Parking Lot 3	2	left	3

**Table 3:** Absolute Trajectory Error (ATE) results and Final Parking Localization Error (FPLE) in the comparison experiments. RMSE means root mean square error. FPLE refers to the Euclidean distance between the estimated and ground truth pose of the vehicle after the parking is completed. PF denotes the Particle-Filter-based Semantic Visual SLAM [25]. The minimum values for both environments are small and are not specified in the table.

Case	Method	ATE Max [m]	ATE Mean [m]	ATE RMSE [m]	FPLE [m]
**1**	PF	0.667	0.326	0.378	0.647
	Modified	0.421	0.164	0.193	0.285
	Proposed	0.299	0.107	0.124	0.137
**2**	PF	0.954	0.284	0.410	0.851
	Modified	0.431	0.239	0.259	0.155
	Proposed	0.362	0.150	0.174	0.056
**3**	PF	1.253	0.485	0.545	1.017
	Modified	0.563	0.250	0.271	0.482
	Proposed	0.394	0.183	0.201	0.394
**4**	PF	0.802	0.222	0.296	0.790
	Modified	0.807	0.334	0.407	0.613
	Proposed	0.446	0.132	0.169	0.224
**5**	PF	0.941	0.413	0.459	0.771
	Modified	0.784	0.317	0.373	0.572
	Proposed	0.423	0.142	0.161	0.198
**6**	PF	0.686	0.260	0.308	0.686
	Modified	0.563	0.282	0.320	0.509
	Proposed	0.301	0.164	0.201	0.270
**7**	PF	0.830	0.245	0.328	0.648
	Modified	0.513	0.230	0.257	0.361
	Proposed	0.300	0.159	0.174	0.121
**8**	PF	0.708	0.295	0.349	0.688
	Modified	0.561	0.332	0.378	0.495
	Proposed	0.235	0.102	0.111	0.234
**9**	PF	0.865	0.265	0.312	0.470
	Modified	0.457	0.193	0.214	0.443
	Proposed	0.388	0.161	0.189	0.379
**10**	PF	1.047	0.537	0.602	1.005
	Modified	0.492	0.173	0.212	0.427
	Proposed	0.430	0.143	0.175	0.416

Note. Cases 1–5 Video Link: https://youtu.be/ch6ISRgMesw (accessed on 8 September 2023). Cases 6–10 Video Link: https://youtu.be/vT5p8-7-PdI (accessed on 8 September 2023).

## Data Availability

The generated data for experiment is available via the following link: https://github.com/ywlee0305/Accurate-Visual-SLAM-against-AVM-Distortion-Error-using-Weighted-GICP-Dataset/tree/master/Training_Data (accessed on 9 July 2023).
